# Cohesive Gold for Filling

**Published:** 1893-02

**Authors:** C. H. Garrish


ARTICLE V.

### COHESIVE GOLD FOR FILLING.

From an ingot of pure gold, how can we make cohesive, semi cohesive and non-cohesive foil ?

We are well informed of the fact that cohesive foil loses its wielding properties if long exposed to the air, or by gases or other impurities.* Heat expels them and re-

---

*** Dr. Watt, maker of the Crystal gold, once showed me some of his gold that had been four years exposed, that was soft and cohesive still.—ED. "Items."** 

stores the original properties, but to what treatment can we subject soft foil to increase its non-cohesiveness? Is it a trade secret?

In the order of merit I consider "gutta-percha" the very best for doubtful and frail teeth. Next comes *tin foil*, then soft gold foil, and, last in the list, cohesive and the many forms of gold which require wielding in their manipulations, while the plastic fillings, such as the oxychloride and phosphates, are uneven or uncertain in their results, yet, nevertheless, indispensable, while amalgams come in to cover for us a multitude of sins, tiding over many a troubled sea, and saving more teeth than any one of the other materials, and possibly more than *all* of them.

For the present I would ask your attention to some advantage of soft gold. Hill's stopping and tin foil respond too quickly to the wear and tear of mastication. Why does the farmer plug the tap-hole of his cider-barrel with a spile made of pine rather than hard wood? Simply because it makes no leakage. For the same reason a boat-builder stops up the holes made in his planks by the withdrawing of nails or screws with soft wood.

The plug must be softer than the material into which it is driven. When you put a soft foil filling into a tooth, you have these same conditions present. You will agree with me that it is not the most solid filling that preserves the tooth, but rather the one that is the best adapted to the inequalities of the cavity, especially the marginal walls, —the one which excludes air, moisture, and germs, and includes sufficient hardness to withstand mastication.

In all these requirements soft gold stands foremost. It is impossible to make as hard a filling as with the cohesive, though it will be dense. It is like putty, though you work it ever so long, when you have finished your labors it is putty still; you have not changed the character of the material, Again, the arrangement of the cylinders in a soft filling is more conducive to a perfect stop.

Will you bear with me while I describe briefly my method of preparing and working the foil? I use Abbey's foil, Nos. 3 and 4, nothing heavier. Take a sheet and fold the edges together, once, twice, thrice, smoothly, making a ribbon of eight thicknesses of foil, about one-half inch wide, then roll or twist into a coil or rope, being careful to keep the surface of the foil smooth. Now with scissors cut into pieces just long enough to suit the cavity to be filled. By that I mean that one end of the coil shall touch the bottom, the other projecting just beyond the orifice. With your tweezers take up a piece of foil and carry it into one corner or angle of the cavity, cut end down, so the coil shall stand on end, condensing toward the distant wall. Using the side of your plugger, another piece is placed alongside, and still another, till you reach the other angle. The size of the cavity is now reduced. Repeat this operation till your last coil is in position. Now with the point of the plugger condense the surplus gold projecting above the tooth, keeping it well over the cavity. This is important. In condensing the gold you will find the weak places in the plug. Send the instrument well to the bottom of the cavity, using lateral wedging pressure. Fill up this pit and look for another. If possible, make these pits a little way from the enamel of walls, so as not to mar or grind the tooth under the instrument. Each piece of foil introduced in this way acts on the filling as the keynote to the arch, the filling being a series of arches. After this is gone through with to your satisfaction, use your burnisher, and use it thoroughly.

What becomes of it? Every piece of gold presents its edge or end to the action of the burnisher (shall I liken the filling to a bunch of asparagus standing on end?), and the action of the burnisher has forced, swaged, molded, or moved the mass in the same manner, but to a less degree, "than the warm burnisher does the gutta-percha plug," bulging the gold toward the walls of the cavity, filling up every inequality and securing for you a perfect stopping.

I start the filling with soft foil, working in the manner I have described, only taking more time to condense the gold as the use of the burnisher, which I emphasize so strongly, is prohibited; therefore the plugger is used a longer time.

A sheet of No. 3 cohesive foil is cut into four pieces, these pieces rolled into coils, each coil cut, making two or three short ribbons; pass one of these ribbons through the blaze of an alcohol lamp, taking care not to burn the foil. Some operators make a mistake in the use of heat. The less used, and sufficient to work your foil, the better. With the foil-carriers set it against the plug, and with rather a coarse instrument carry it across one edge of the filling; then, with a fine-pointed instrument, work it persistently; retrace your steps; add another coil, working on the former cohesive part, and overlap onto the soft filling slightly. The advantage of this method is a perfect weld. I have no difficulty in welding cohesive foil onto a soft filling, and never make a filling entirely of cohesive foil.

Soft foil can be used with a minimum loss of tooth substance, especially in proximal cavities of the incisors. I believe in the free use of files and chisels. Soft gold demands it, but the manner of working it by wedging enables the operator to fill without cutting a direct opening to the cavity.

The soft foil may be abused, yet can hardly change its character; but not so with cohesive; it responds quickly and resents every abuse. Virtue goes out of it at the first, and these conditions require a generous opening or channel, and often that is made from the labial surface. This result, while it caters to the vanity of a few patients, filling the heart of the operator with pride, is poor taste.

I do not think the art of filling teeth with soft foil an easy one to attain for it requires much time and practice, and the results are not so pleasing to the eye. In appearance they do not compare with cohesive work, but they have one strong virtue, they are honest, even better than they look, as the fillings of Drs. Wetherbee, Pray and Johnson, done thirty, yes, forty, years ago, show to be. I have reached the conclusion that the man himself is greater than his school or methods, but I ask you not to let go of all the ancient landmarks. Instruct your students in the art of making soft foil fillings; there is merit in them. They are to dentistry what bread is among our food supplies.—C. H. GARRISH, in *International.*

